# CD4 count recovery and associated factors among individuals enrolled in the South African antiretroviral therapy programme: An analysis of national laboratory based data

**DOI:** 10.1371/journal.pone.0217742

**Published:** 2019-05-31

**Authors:** Tendesayi Kufa, Zara Shubber, William MacLeod, Simbarashe Takuva, Sergio Carmona, Jacob Bor, Marelize Gorgens, Yogan Pillay, Adrian Puren, Jeffrey W. Eaton, Nicole Fraser-Hurt

**Affiliations:** 1 Centre for HIV and STIs, National Institute of Communicable Diseases, Sandringham, Johannesburg, South Africa; 2 The School of Public Health, Faculty of Health Sciences, University of the Witwatersrand, Johannesburg, South Africa; 3 The World Bank Group, Washington D.C., United States of America; 4 Department of Global Health, Boston University School of Public Health, Boston, United States of America; 5 Health Economics and Epidemiology Research Office (HE^2^RO), Department of Internal Medicine, School of Clinical Medicine, Faculty of Health Sciences, University of the Witwatersrand, Johannesburg, South Africa; 6 Perinatal HIV Research Unit, Faculty of Health Sciences, University of the Witwatersrand, Johannesburg, South Africa; 7 National Priority Programmes, National Health Laboratory Services, Sandringham, Johannesburg, South Africa; 8 National Department of Health, Pretoria, South Africa; 9 Division of Virology, School of Pathology, Faculty of Health Sciences, University of the Witwatersrand, Johannesburg, South Africa; 10 Department of Infectious Diseases Epidemiology, Imperial College, London, United Kingdom; University of KwaZulu-Natal, SOUTH AFRICA

## Abstract

**Background:**

We describe CD4 count recovery among HIV positive individuals who initiated antiretroviral therapy (ART) with and without severe immune suppression using complete laboratory data from South Africa’s national HIV treatment programme between 2010 and 2014 and discuss implications for CD4 count monitoring.

**Methods:**

Retrospective analysis of routinely collected laboratory data from South Africa’s National Health Laboratory Service (NHLS). A probabilistic record linkage algorithm was used to create a cohort of HIV positive individuals who initiated ART between 2010 and 2014 based on timing of CD4 count and viral load measurements. A CD4 count < 50 copies/μl at ART initiation was considered severe immunosuppression. A multivariable piecewise mixed-effects linear regression model adjusting for age, gender, year of starting ART, viral suppression in follow up and province was used to predict CD4 counts during follow up.

**Results:**

1,070,900 individuals had evidence of starting ART during 2010–2014 and met the criteria for inclusion in the cohort -46.6% starting ART with CD4 < 200 cells/μl and 10.1% with CD4 < 50 cells/ μl. For individuals with CD4 counts < 200 cells/μl, predicted CD4 counts > 200 cells/μl, >350 cells/μl and >500 cells/μl corresponded with mean follow up durations of 1.5 years (standard deviation [s.d] 1.1), 1.9years (s.d 1.2) and 2.1 years (s.d 1.3 years). For those with CD4 counts < 50 cells/μl, predicted CD4 count above these threshold corresponded with mean follow up durations of 2.5 years (s.d 0.9 years), 4.4 years (s.d 0.4 years) and 5.0 years (s.d 0.1years) for recovery to the same thresholds. CD4 count recovery varied mostly with duration on ART, CD4 count at the start of ART and gender.

**Conclusion:**

For individuals starting with ART with severe immunosuppression, CD4 recovery to 200cells/μl did not occur or took longer than 12 month for significant proportions. CD4 monitoring and interventions recommended for advanced HIV disease should continue until full recovery.

## Introduction

Antiretroviral therapy (ART) reduces mortality and morbidity among HIV positive individuals as well as the onward transmission of HIV. [1–3] Untreated HIV infection is associated with decreases in CD4 count levels and increases in HIV plasma viral loads. [4]The initiation of ART is typically followed by declines in viral loads and increases in CD4 count. CD4 count recovery following ART initiation is rapid in the first few months, as a result of redistribution of the existing CD4 cells from lymphoid organs, and then slows down as new CD4 cells are made as a result of thymic activation.[5,6] A number of factors are known to affect the extent of CD4 count recovery post ART initiation. These include CD4 count at ART initiation, gender -with males found to have lower CD4 recovery compared to females in some settings, [7] older age, [8,9] duration on ART and ART regimen, [10] as well as genetic and environmental factors that contribute to immune activation.[6]

Because the CD4 count is a good predictor of morbidity and mortality before and after ART start, HIV treatment guidelines in the past recommended CD4 count measurements for determining eligibility for HIV treatment and for monitoring its effectiveness. Poor or suboptimal CD4 recovery despite ART and viral suppression has been associated with increased risk of mortality and morbidity from tuberculosis and other AIDS related morbidities.[11–15] Since the introduction of the universal test and treat strategy and removal of CD4 count based criteria for initiating ART, as well as the increasing availability of VL monitoring technologies, there has been ongoing discussions about the role of CD4 count measurement prior to and after ART initiation.[16,17] Advocates of discontinuing baseline CD4 count measurement cite its limited utility in predicting high viral load, disease progression or risk of high transmission as well as its cost and its variability in the same individuals as possible reasons why it should not be measured at or before ART start. In addition, there are little or no changes to CD4 count levels among virally suppressed individuals on long term ART.[17] On the other hand, advocates of maintaining CD4 count measurements argue that there are still significant proportions of HIV positive individuals who present with advanced HIV disease who need to be identified for prophylaxis against opportunistic infections and intensive monitoring for immune recovery.[18–22] Also, in the context of suboptimal viral suppression and development of ARV drug resistance, individuals may experience CD4 declines necessitating CD4 count- guided management such as cotrimoxazole or fluconazole prophylaxis.[22] We used a large national laboratory-based database and probabilistic matching algorithms to describe the timing and magnitude of CD4 count recovery over a period of up to 60 months among HIV positive individuals who initiated ART in the South African national HIV treatment programme between 1^st^January 2010 and 31^st^ December 2014 and followed up until March 2015. We discuss the implications of the findings on the continued use of CD4 count measurements to monitor the effectiveness of ART.

## Methods

Some of the methods of this paper have been described elsewhere [23]. Briefly the South African government has been providing ART at public health facilities through the comprehensive care, management and treatment (CCMT) programme since 2004. At inception of the programme, HIV positive individuals with CD4 counts <200 cells/μl OR those with WHO stage III/IV were eligible for ART while CD4 count and viral load monitoring was conducted at baseline and every six months.[24] By the end of 2016, the guidelines recommended that all HIV positive individuals be started on ART irrespective of CD4 count, that the CD4 count be measured at ART initiation and at 12 months only and that VL be measured at 6 months, 12 months and annually thereafter.[25,26] The growth of the CCMT programme over this time has been exponential, from a few NGO supported ART sites in 2004 to 3775 public sector facilities in the country and supported by the National Health Laboratory Service’s (NHLS) 60 CD4 count and 17 viral load laboratories.[27]

Routine specimen data from the laboratory management information system are archived in the Corporate Data Warehouse (CDW) of the NHLS and are available for analysis. However, the lack of a unique identifier for individuals enrolled into the CCMT programme and the inability to uniquely identify multiple tests over time and treatment sites has limited the use of these data in the past. As part of a broader evaluation of adherence and retention in HIV care within the South African National ART programme, the National Department of Health commissioned analyses of laboratory datasets using big data methods in 2015. Initial analyses of these data describing i) proportions of individuals who had CD4 recovery to given thresholds, ii) the time to recovery to these threshold and iii) speed of CD4 recovery in the first 12 months were conducted in 2015–2016 and have been published elsewhere. [23] The resulting cohort has also been used to describe viral suppression, [27] late presentation [21] and retention in care [28]

### Cohort inclusion and exclusion criteria

The procedures for record linkage and creating a synthetic cohort of HIV positive individuals has also been described in detail elsewhere. [23] For this analysis unique individuals who had i) at least one viral load test result in the CDW database, ii) had at least two CD4 count results in the database and iii) had a CD4 count in the database that was done three to 12 months prior to the first viral load test- considered eligible baseline CD4 count iv) aged 15 years or older at date of eligible CD4 count were included. Based on national guidelines stipulating a baseline CD4 count measurement at the time of ART initiation and VL measurement 6 months after initiation, the dates of CD4 measurement occurring three to 12 months prior to the first VL were used to determine the estimated ART start date for individuals who were likely to have started ART during this time (2010–14). This period was selected because prior to 2010, not all provinces archived data in the CDW and in 2015, the ART monitoring guidelines changed, removing the 12 month CD4 count measurement for patients who did not have advanced disease at ART start. Data on these eligible individuals were used to construct a cohort, with cohort entry at ART start and the date of the eligible baseline CD4 count being used as a proxy for the date of ART start.

### Variables, definitions and outcomes

The date of ART start was defined as the date of the eligible baseline CD4 count described above. This date was taken to be the date of a CD4 count done three to 12 months before the first viral load date. Where multiple CD4 count test dates and results met this criterion, the earliest date was used. The CD4 count at ART start was defined as the result of this eligible baseline CD4 count while follow up CD4 counts were results from the CD4 count tests done at any point after the ART start date. The duration of follow up was defined as the time interval between the date of ART initiation and the date of the latest CD4 count and was considered to be the same as the duration on ART assuming that individuals remained on ART for the entire duration of follow up. A CD4 count of < 50 cells/μl at the start of ART was considered severe immunosuppression at ART start. Viral suppression was defined as having a viral load result of <400 copies/ml at any point during follow up while age referred to the individual’s age recorded at the eligible baseline CD4 count or determined from the recorded date of birth. Gender and year of ART initiation was taken to be the gender and year recorded at the eligible baseline CD4 count testing. Province was defined as the province where eligible baseline CD4 count testing was done regardless of whether or not the individual subsequently moved to other provinces. The main outcome of the analysis was the mean CD4 counts at given intervals post ART initiation. For individuals who had multiple CD4 counts done in the same interval of follow up, all measurements were used in determining the mean CD4 count in that interval.

### Statistical analysis

Once the data were linked to unique individuals using the linkage algorithm, it was exported to Stata 14.2 (Stata Corp, College Station, Texas, USA) for analysis. The characteristics of eligible individuals at cohort entry were described using medians and interquartile ranges for continuous variables and as frequency and proportions for categorical data. A multivariable piecewise mixed-effects linear regression model—adjusting for age, gender, eligible baseline CD4 count, any virological suppression during follow up and province with splines at 3, 6, 12, 24, 36, 48 and 60 months post ART initiation—was used to predict the mean square root of CD4 counts during and at specified time intervals. The model was fit on the square root scale for the CD4 count outcome in order to normalise the distribution of the CD4 counts. The model allowed for the following interactions: i) age and gender, ii) eligible baseline CD4 count and duration on ART (as splines), iii) calendar year and duration on ART, iv) gender and duration on ART and v) province of entry into care and duration on ART. Predicted mean CD4 counts at different time intervals post ART start and for different combinations of age, gender and baseline CD4 count were obtained by squaring the predicted square roots of the CD4 counts. Likelihood ratio tests comparing i) the piecewise regression model with splines at 3, 6, 12, 24, 36, 48 and 60 months post ART initiation with no covariates (representing observed data) to the full model and ii)the same piecewise regression model but with no interaction terms to the full model were used to determine goodness of fit.

### Ethical considerations

This study was approved by the University of the Witwatersrand Human Research Ethics Committee (HREC) and the Boston University School of Medicine IRB (H-31968.) Permissions were also obtained from the NHLS Research Office and the National Department of Health. Since this was a secondary analysis of routine laboratory data, no written informed consent was obtained from patients.

## Findings

### Description of the population

**Fig 1:**
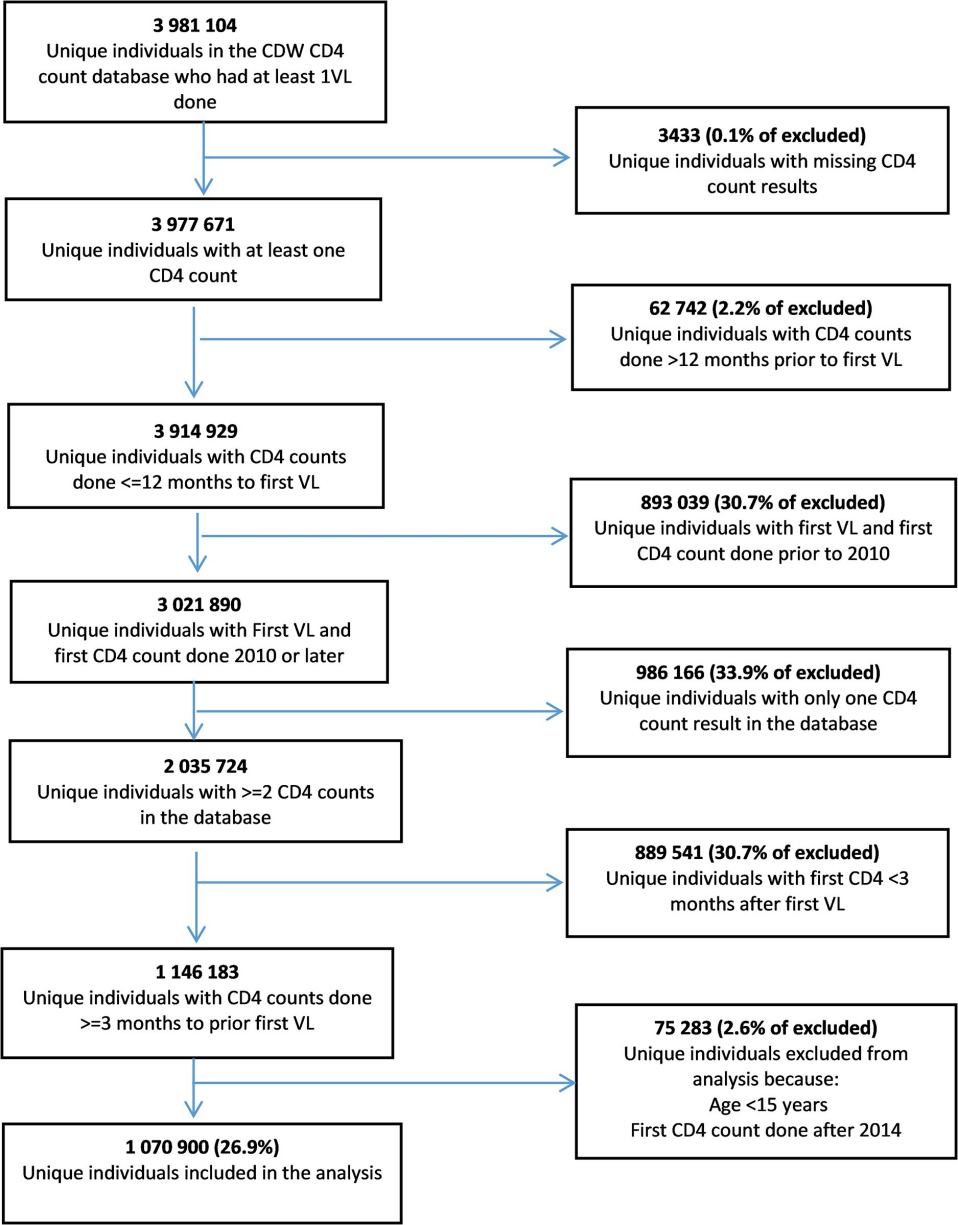
Study Flow.

From 3,977,761 unique individuals who had at least one viral load test and one CD4 count test record in the CDW database between January 2010 and March 2015, 1 070 900 (26.9%) individuals met the criteria for inclusion in the cohort. Fig 1 shows the numbers of individuals who were excluded from the analysis and the reasons for the exclusions. The demographic and CD4 count profiles of those included in the analysis have been presented elsewhere (23). In summary; of those included, 321 945 (30.1%) were male, 17.7% were aged 35 years or younger, median baseline CD4 count was 213cells/μl (IQR 117–324) with approximately 10% of individuals having CD4 counts < 50 cells/μl, median duration of follow up was 24 months (IQR 12.2–36.9 months) and the median number of CD4 measurements in the database (including the eligible baseline CD4 count) was 3 (IQR 2–4)–see Table 1.

**Table 1:** Description of population included in the analysis (N = 1 070 900).

Variable	
Male (n, %)*	321 945 (30.1)
Age distribution, (n, %)	
15–24	11 521 (1.1)
25–34	172 009 (16.1)
35–49	562 693 (52.5)
50+	324 677 (30.3)
Year of starting of ART, (n, %)	
2010	212 906 (19.9)
2011	263 824 (24.6)
2012	255 998 (23.9)
2013	228 555 (21.4)
2014	109 617 (10.2)
CD4 count at baseline, Median (IQR)	213 (117–324)
Distribution of CD4 counts baseline, (n, %)	
<50	107 706 (10.1)
50–199	391 076 (36.5)
200–349	358 258 (33.5)
350–499	114 885 (10.7)
> = 500	98 972 (9.2)
Duration of follow up in months, Median (IQR)	24 (12.2–36.9)
Virally suppressed in follow up (n, %)	919 649 (85.9)
Duration of follow up (n, %)	
<12	258 964 (24.7)
12–24	276 028 (25.8)
24–35	246 600 (23.0)
36–47	189 271 (17.7)
> = 48	100 037 (9.3)
Province (n, %) **	
EC	109,615 (10.2)
FS	62,785 (5.9)
GP	223,080 (20.8)
KZN	341,180 (31.9)
LP	66,567 (6.2)
MP	92,660 (8.7)
NC	17,451 (1.6)
NW	65,585 (6.1)
WC	90,105 (8.4)

*gender information missing for 16587 (1.6%),

** province information missing for 1872 (0.2%)

Compared to individuals included in the cohort, individuals excluded from the cohort were more likely to be male (33.3% vs. 30.1%), older (median 46 years [IQR 38–55 years] vs. 44 years [IQR 37–52 years]), had a higher median nadir (lowest ever) CD4 count (200 cells/μl vs. 167 cells/μl) and were less likely to be from KwaZulu-Natal Province (22% vs. 31.9%). Across all provinces the median CD4 counts at the start of ART were lower among males compared to females, decreased with increasing age from age category 25–34 years and increased with calendar years from 2010. [23]

### Observed CD4 count recovery

**Fig 2:**
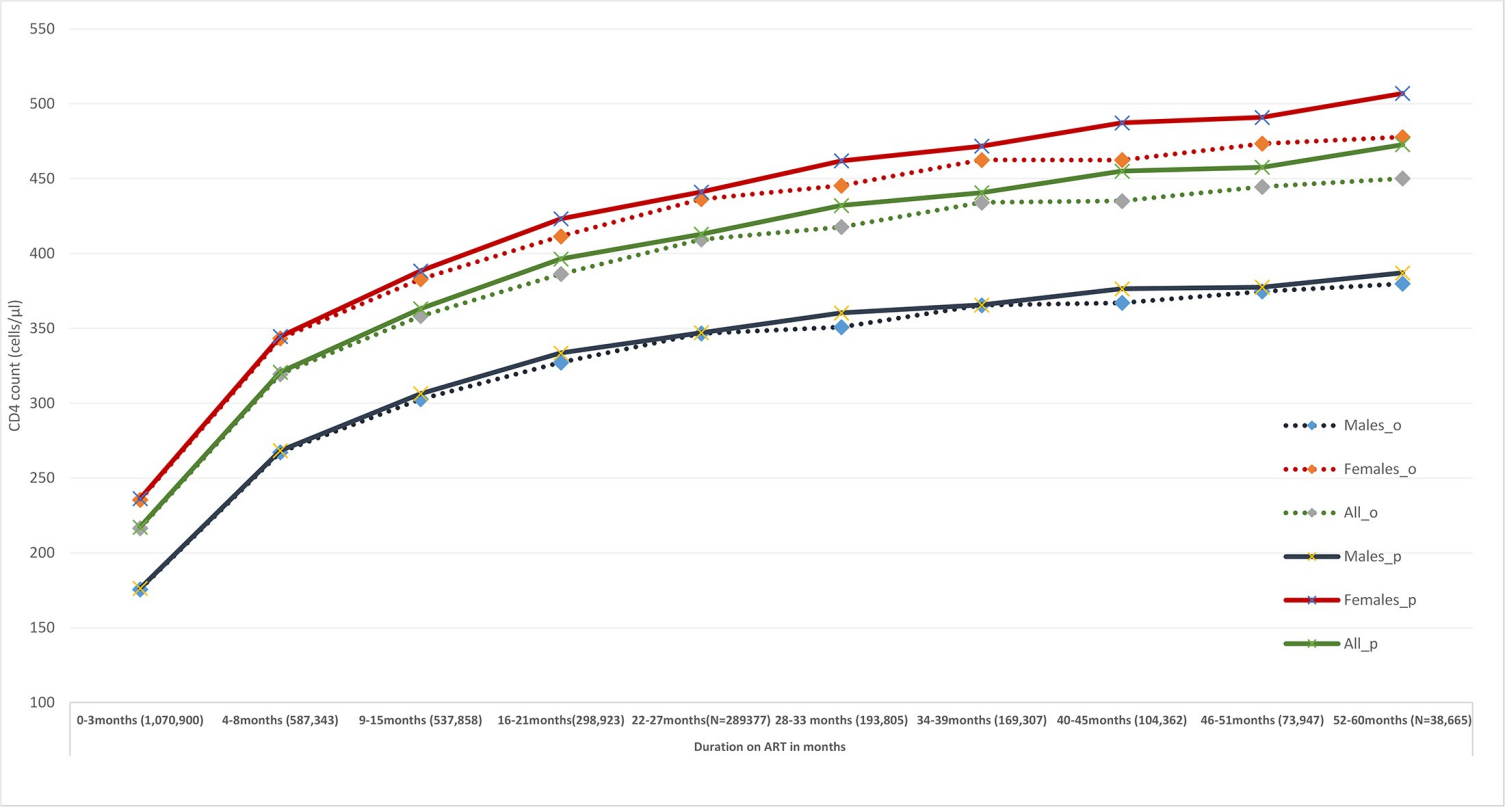
Observed and predicted CD4 counts at different durations on ART.

Overall the observed CD4 counts increased rapidly from baseline to a mean of 319 cells/μl (standard deviation [s.d] 36, median 315 (IQR 198–463 cells/μl)) around 6 months post ART initiation. Beyond that, there were smaller but steady increases in the observed means to 358 cells/μl (s.d 36 cells/μl) at around 12 months, 409cells/μl (s.d 37 cells/μl) at 24 months, 434 cells/μl (s.d 40cells/μl) at 36 months, and 544 cells (s.d 44 cells/μl) by around 48 months post ART initiation. At all the time intervals, the observed CD4 count levels were lower among males compared to females (see Fig 2 and S1 Table).

### Overall predicted increases in CD4 counts during follow up

**Fig 3:**
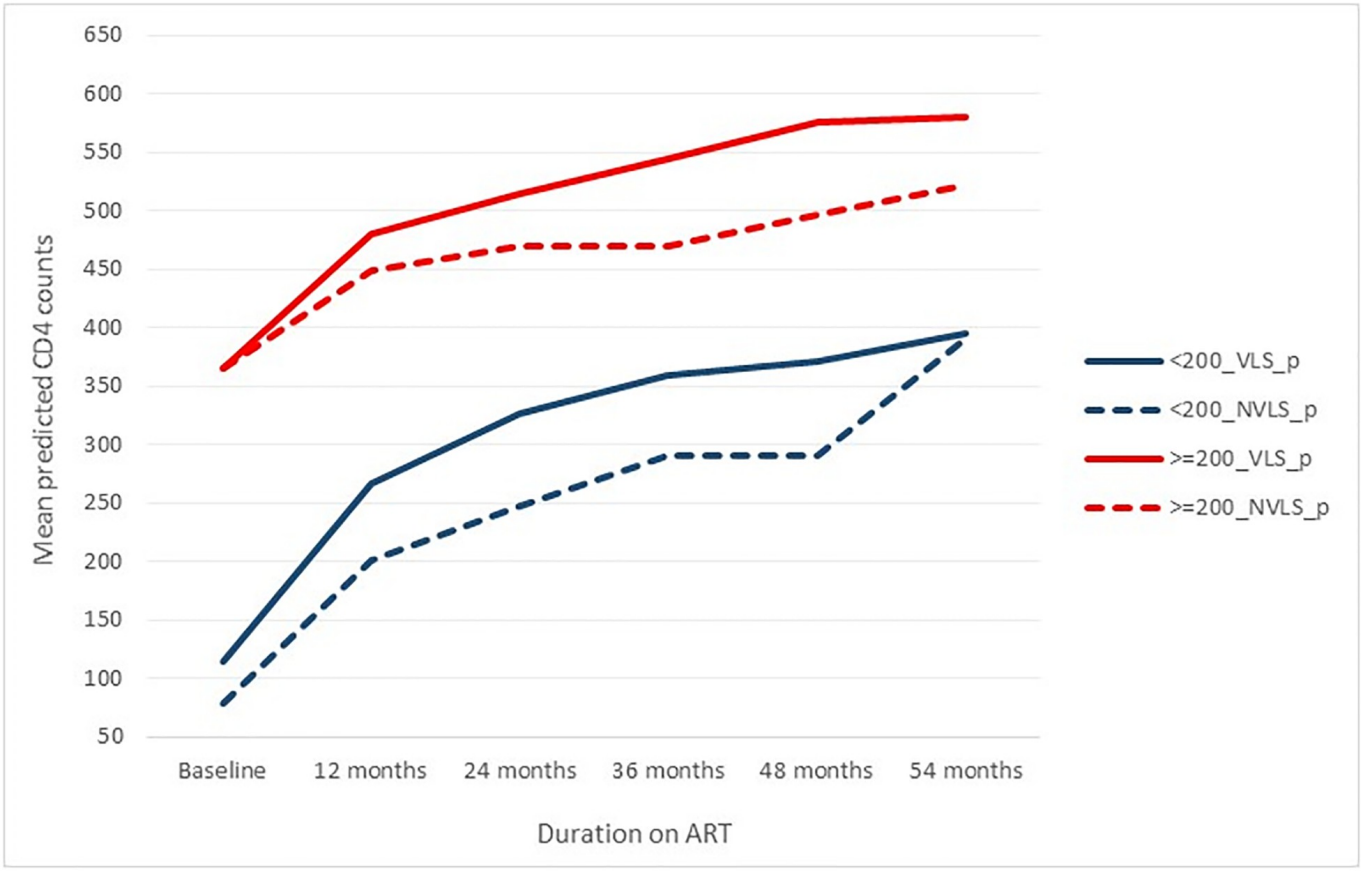
Predicted CD4 counts among those with or without viral suppression during follow up.

Overall the predicted mean CD4 recovery was rapid during the first eight months on treatment before slowing down in the subsequent periods. The mean predicted CD4 counts during the first three months was 217 cells/μl (95% Confidence intervals [CI] 216–218 cells/μl). The mean predicted CD4 counts increased to 321 cells/μl (95% CI 319- 322cells/μl around 6 months; 363 cells/μl (95% CI 362–365 cells/μl) around 12 months; 396 cells/μl (95% CI 395–398 cells/μl) around 18 months; 413 cells/ μl (95% CI 411–415 cells/μl) around 24 months; 432 cells/μl (95% CI 430–435 cells/μl) around 30 months; 441 cells/μl (95% CI 437–444 cells/μl) around 36 months; 455 cells/ μl (95% CI 452–459 cells/μl) around 42 months; 458 cells/μl (95% CI 453–463 cells/μl) around 48 months and 473 cells/μl (95% CI 466–480 cells/μl) around 54 months. For individuals with CD4 counts < 200 cells/μl; predicted CD4 counts >200 cells, 350 cells/μl and 500 cells/μl corresponded with a mean ART durations of 1.5 years (s.d 1.1), 1.9 years (s.d 1.2) and 2.1 years (s.d 1.3 years) respectively. At all durations on ART, the predicted recovery was lower among males compared to females (Fig 2 and S1 Table) and among those who did not achieve viral suppression at any point during follow up (Fig 3 and S2 Table). Qualitatively the mean predicted CD4 counts were close to the mean of the observed CD4 counts throughout all the durations on ART among males while for females the predicted means were increasingly higher among females at longer durations, starting at 9–12 months post ART initiation. However the full model with interactions was different to the model without any covariates (observed data)—likelihood ratio test p<0.001- and also from the model without interactions between covariates and duration on ART -likelihood ratio test p<0.001.

### Predicted CD4 count recovery for individuals with CD4 counts< 50 cells/μl at baseline

For individuals who started ART with CD4 counts < 50 cells/ μl—severe immunosuppression, the mean predicted CD4 count at 12 months was 177 cells/μl (95% CI 175–179 cells/μl) overall. This mean was higher among females compared to males [192 cells/μl (95% CI 190–193 cells/μl) vs 160 cells/μl (95% CI 158–161 cells/μl)]. For both males and females, the mean predicted CD4 counts at 12 months were <200 cells, consistent with the observation that 71% of this population had not recovered to 200 cells/μl by 12 months of follow up with 39% not achieving CD4 recovery to 200cells/μl at all during follow up. Predicted CD4 counts >200 cells/μl, 350 cells/μl and 500 cells/μl corresponded with ART durations of 2.5 years (S.D 0.9 years), 4.4 years (S.D 0.4 years) and 5.0 years (S.D 0.1years) respectively. Table 2 shows the mean predicted CD4 counts at given durations on ART for males and females.

**Table 2:** Predicted CD4 counts among individuals with CD4 counts < 50 cells/μl at start of ART at different time points during follow up (n = 107 706).

Time point	Predicted CD4 count males (cells/μl, 95% CI)	Predicted CD4 count females (cells/μl, 95% CI)	Predicted CD4 count all (cells/μl, 95% CI)
12 months	160 (158–161)	192 (190–193)	177 (175–179)
24 months	213 (211–215)	262 (260–264)	238 (236–240)
36 months	240 (238–243)	301 (298–305)	273 (270–277)
48 months	254 (250–259)	323 (318–328)	293 (289–299)
54 months	252 (245–261)	324 (318–331)	291 (284–298)

### Predicted CD4 count recovery among older individuals (age ≥50 years)

Because CD4 counts at the start of ART decreased with increasing age, the predicted CD4 count recovery among the oldest group of individuals was also determined at the given time points and for given baseline CD4 counts. Among the 324,667 individuals who were aged ≥50 years at the start of ART, the predicted CD4 counts at 12, 24, 36, 48 and 54 months increased with CD4 counts at the start of ART and were higher among females compared to males. For males with CD4 counts <50 cells/μl, the predicted mean of the CD4 count peaked at 48 months at 254 cells/μl while for females it continued to increase to 325 cells/μl by 54 months. For those with CD4 counts ≥500 cells/μl, the predicted CD4 count peaked at 54 months for males and 60 months among females at 612 cells/μl and 705 cells/μl respectively (see S3 Table). Compared to males ≥50 years of age with CD4 counts < 50 cells/μl at ART start who had mean CD4 recovery to 159 cells/μl (95% CI 158–161 cells/μl) at 12 months post ART initiation, males aged 15–24 years, 25–34 years and 35–49 years old with similar CD4 counts at ART start had CD4 count recovery to 157 cells/μl (95% CI 156–160 cells/ μl), 176 (95% 175–177 cells/μl) and 157 cells/μl (95% 155–158 cells/μl) respectively,showing no clear decrease in extent of CD4 recovery with increasing age. On the other hand, females aged 15–24 years, 25–34 years and 35–49 years who had CD4 counts < 50 cells/μl at ART start had predicted CD4 counts of 200 cells/μl (198–203 cells/μl), 198 cells/μl (196- 199cells/μl) and 191 cells/μl (190–193 cells/μl) at 12 months post ART initiation respectively compared to 188 cells/μl (186–189 cells/ μl) among females ≥50 years, demonstrating decreasing CD4 recovery with increasing age.

### Predicted CD4 count recovery across provinces

To compare the extent of CD4 count recovery across the nine provinces in the country, the predicted CD4 counts at 12, 36 and ≥54 months were determined for males and females aged 15–49 years with CD4 counts 50–199 cells/μl and ≥200 cells/μl at ART start. Among males, the mean predicted CD4 counts at 12, 36 and ≥54 months for individuals with CD4 counts of 50–199 cells/μl at ART start and for those with CD4 counts ≥ 200 cells/μl were highest in KwaZulu Natal, Free State and Gauteng provinces and lowest in the Eastern Cape, Northern Cape and North West provinces (see S4 Table). Similar trends were observed among females with the mean predicted CD4 counts at 12, 36 and > 54 months also highest in KwaZulu Natal, Free State and Gauteng and lowest in Northern Cape, Eastern Cape and North West provinces (see S5 Table). However, despite the provincial differences in CD4 recovery, CD4 count at the start of ART was a stronger independent determinant of predicted CD4 counts at the different ART durations periods than province.

### Predicted CD4 count recovery by calendar year

As median CD4 counts at the start of ART increased with calendar year, [23] an analysis of CD4 count recovery at 12 months among all individuals initiating ART over the five calendar years 2010 to 2014 was also conducted. Across all categories of CD4 count at ART start, the predicted mean CD4 counts at 12 months dipped slightly in 2011 but otherwise remained stable over the calendar years (S1 Fig and S6 Table). CD4 count at the start of ART was a stronger determinant of predicted CD4 counts than the calendar year of ART start.

## Discussion

We used a multivariable piecewise mixed-effects linear regression model adjusting for age, gender, eligible baseline CD4 count, virological suppression during follow up and province to predict the extent and magnitude of CD4 count recovery among individuals aged >15 years old initiating ART. Our analyses found that across all ages, provinces and baseline CD4 counts, CD4 recovery was lower and slower among males compared to females even after adjusting for CD4 count at ART start as well as duration on ART. The analysis also found that among individuals with CD4 counts <50 cells/μl at the start of ART, the predicted mean CD4 count after 12 months post ART initiation remained below 200 cells/μl and that it took a mean of 2.5 years for recovery to 200 cells/μl in this group. Our analyses also consistently found greater CD4 count recovery in KwaZulu Natal, Gauteng and Free State provinces.

Although this is the first ever national level analysis of CD4 count recovery data, this analysis confirms what smaller cohorts of up to 90 000 individuals from South Africa and other sub Saharan African settings have demonstrated. These studies demonstrated that i) the CD4 count at the start of ART was the strongest predictor of CD4 count recovery, [29] ii) CD4 recovery was rapid early in the course of ART but stabilised around 12 months post ART initiation, [30] and iii) CD4 count recovery is slower in males compared to females and in older individuals compared to younger individuals. [7,8,31,32] However, the comparison of CD4 count recovery by province and calendar year is new. A number of factors may contribute to the variation in CD4 counts across provinces even after adjusting for confounding factors such as age, gender, baseline CD4 counts and viral load suppression. Genetic and socio-economic factors may affect immune recovery. An international study including data from the IeDEA cohort demonstrated poorer immune recovery in East Africa compared to other regions of Africa and the world after adjusting for ARV regimen differences and background CD4 count variations.[33] The effect of calendar year remained significant even after adjusting for increasing CD4 counts at ART start and could be explained by changes in some other unmeasured characteristics of the CCMT programme such as the quality of HIV care provided or regimen changes.

Our study had some important strengths. Firstly this is a national level data set with representation of all provinces in the country. Second, there were long durations of follow up—up to five years for some eligible individuals. This meant the trajectories of CD4 count recovery could be described beyond one to two years. Our study also had a number of limitations: Firstly, this analysis used laboratory data linked to unique individuals through the use of a probabilistic matching algorithm to create a cohort and determine CD4 recovery outcomes. The accuracy of the cohort data depended on the performance of the algorithm used to link CD4 count and viral load data to unique individuals, and on the completeness of data in the database. The algorithm used to construct the cohort had a sensitivity of 89% and a positive predictive value of 85%, which means it matched correctly for the majority of individuals in the cohort. [27] It is therefore unlikely that the performance of the matching algorithm affected our conclusions. Secondly, the eligibility criteria required that unique individuals have an estimated ART start date between 2010 and 2014, have a CD4 count done 4–12 months prior to the first viral load and have at least two CD4 count measurements. These three criteria accounted for the majority of the unique individuals in the laboratory database being ineligible for inclusion in the analysis with the resultant cohort including only 27% of those in the initial database. Individuals with only one CD4 count reading may have died or been lost to follow up, outcomes which are both associated with poor CD4 count recovery. This may have resulted in selection bias and may limit the extent to which these findings can be generalised to all individuals enrolled in the CCMT programme in South Africa. The comparison of demographic and clinical characteristics of those excluded to those included suggests that those excluded would likely have had lower and slower recovery rates as they were more likely to be male and older, although they had higher nadir CD4 counts. [23]Thirdly, guidance on the frequency of CD4 count monitoring changed between 2010 and 2014. From 2010–2011, CD4 count measurements were taken at ART initiation, at 6 months, 12 months, and annually thereafter; whereas from 2012 onwards, CD4 count measurements were taken at ART initiation then at 12 months and annually or as indicated thereafter. This meant that fewer individuals in the later years had a baseline CD4 count and at least one other CD4 count. Also, individuals who initiated ART in the latter years were less likely to be included as they had limited follow up time. In addition, the assumptions in the analysis are sensitive to guideline adherence by clinicians. Differences in the extent to which facilities adhered to the guidelines on CD4 count and viral load monitoring could cause variability across provinces and districts with respect to eligibility to enter the cohort and in the number of CD4 counts in follow up. It is known that the proportions of individuals on ART who have viral loads done at 6 months or 12 months varies substantially across districts, sub-districts and facilities.[27] However the mixed-effects regression model used in the analysis is robust to missing data and likely minimised the effects of missing CD4 counts. The model used allowed for the missingness to be correlated with baseline variables and existing trends and therefore multiple imputation or other approaches to handle the missing data was not necessary. Fourth, the actual ART start dates for the cohort were not known but were inferred from the dates of the baseline CD4 counts which were in turn estimated from the date of the first viral load test. We may have overestimated the values of the CD4 count at the start of ART by including people who were already on ART as newly initiated on ART. Fifth, because all the data used came from laboratories, there were no data on deaths or retention in care. The individuals included in this cohort were therefore those who survived and were retained in care long enough to have follow up CD4 count tests done. The absence of data on people who died or had no test results during the study period limits the extent to which the data on CD4 recovery can be used to assess programme performance across provinces. Longer time to recovery may be a positive sign in some cases as it means patients are surviving and included in laboratory monitoring (especially relevant for very late ART initiations). Sixth, we assumed that once individuals started ART they stayed on ART during the entire duration of follow up. Being off ART is associated with loss of viral suppression and poor CD4 count recovery. Last, there was wide variation in the numbers of eligible individuals across provinces.

Despite these limitations, our findings have important implications for policy and practice. CD4 count measurement at entry or re-entry into care should be continued in order to identify individuals with advanced disease and to allow clinicians to define the appropriate package of care. However, ART initiation should proceed regardless of CD4, removing the risk of CD4 count measurement becoming a cause of pre-treatment loss-to-follow-up. The cost-effectiveness of different CD4 screening and monitoring strategies needed to be considered in order to ensure that CD4 guided HIV care is provided in the era of test and treat. Recent data from the South African care and treatment programme shows that as much as 10% of HIV positives who start ART do so with CD4 counts less than 100 cells/μl despite increase in the median CD4 counts at ART start. [21] For those in care, the current guidance on CD4 count monitoring recommends that CD4 count monitoring be done at 12 months post ART initiation for individuals starting ART with CD4 counts less than 200 cells/μl and as recommended by clinicians thereafter. Our findings suggest that this monitoring maybe required beyond this 12-month milestone and recommendation for practice could be that CD4 count monitoring continues annually until recovery to > 200 cells/μl is achieved. We also recommend that with the introduction of a unique identifier into the South African public health sector and the development of a case-based surveillance platform that links laboratory data with clinic level data, national level analyses be conducted to confirm these findings and identify additional factors that may impact CD4 count recovery in South Africa.

## Supporting information

S1 Fig Predicted CD4 counts at 12 months post ART initiation by baseline CD4 counts and calendar year of ART initiation. (DOCX)

S1 Table Mean predicted CD4 count recovery for all individuals at different durations of follow up (N = 1 070 900). (DOCX)

S2 Table Predicted CD4 counts (with confidence intervals) among those with or without viral suppression (N = 1 070 900). (DOCX)

S3 Table Predicted CD4 count recovery among individuals aged 50 years or older at ART start (N = 324,677). (DOCX)

S4 Table Predicted CD4 recovery at different durations on ART among males 15–49 years by baseline CD4 count and province (N = 192 066). (DOCX)

S5 Table Predicted CD4 recovery at different durations on ART among females 15–49 years by baseline CD4 count and province (N = 542 439). (DOCX)

S6 Table Predicted CD4 counts at 12 months post ART initiation by baseline CD4 counts and calendar year of ART initiation (N = 1 070 900). (DOCX)
