# Supplementary material for: CD4 count recovery and associated factors among individuals enrolled in the South African antiretroviral therapy programme: An analysis of national laboratory based data
Source: PLoS One. 2019 May 31;14(5):e0217742. doi: 10.1371/journal.pone.0217742 (PMC6544279; doi:10.1371/journal.pone.0217742)
Supplement: S1 Fig — (DOCX) [file pone.0217742.s001.docx]

**S1 Fig: Predicted CD4 counts at 12 months post ART initiation by baseline CD4 counts and calendar year of ART initiation**
