# Supplementary material for: CD4 count recovery and associated factors among individuals enrolled in the South African antiretroviral therapy programme: An analysis of national laboratory based data
Source: PLoS One. 2019 May 31;14(5):e0217742. doi: 10.1371/journal.pone.0217742 (PMC6544279; doi:10.1371/journal.pone.0217742)
Supplement: S1 Table — (DOCX) [file pone.0217742.s002.docx]

**S1 Table: Mean predicted CD4 count recovery for all individuals at different durations of follow up (N= 1 070 900)**

|  | | **Duration on ART in months** | | | | | | | | | |
| --- | --- | --- | --- | --- | --- | --- | --- | --- | --- | --- | --- |
|  |  | **0-3months** | **4-8months** | **9-15months** | **16-21months** | **22-27months** | **28-33 months** | **34-39months** | **40-45months** | **46-51months** | **52-60months** |
| **Observed** | Males_o | 176 | 267 | 303 | 327 | 347 | 351 | 366 | 367 | 375 | 380 |
|  | Females_o | 236 | 343 | 383 | 412 | 436 | 445 | 463 | 462 | 473 | 478 |
|  | All_o | 217 | 319 | 358 | 386 | 409 | 418 | 434 | 435 | 445 | 450 |
| **Predicted** | Males_p | 176 (175- 177) | 268 (267- 270) | 306 (305- 308) | 334 (332- 335) | 347 (345- 349) | 360 (358- 363) | 366 (363- 369) | 376 (373- 380) | 378 (373- 382) | 387 (381- 394) |
|  | Females_p | 236 (235- 237) | 345 (343- 346) | 388 (387- 390) | 423 (422- 425) | 441 (439- 443) | 462(460- 464) | 472 (469- 475) | 487 (484- 491) | 491 (486- 496) | 507 (500- 514) |
|  | All_p | 217 (216- 218) | 321 (319- 322) | 363 (362- 365) | 396 (395- 398) | 413 (411- 415) | 432 (430- 435) | 441 (437- 444) | 455 (452- 459) | 458 (453- 463) | 473 (466- 480) |

All_o= observed CD4 counts for all; All_p= predicted CD4 counts for all; Females_o= observed CD4 counts for females; Females_p= predicted CD4 counts for all; Males_o= observed CD4 counts for males; Males_p= predicted CD4 counts for males; lb= lower bound of the confidence interval; ub= upper bound of the confidence interval
