# Supplementary material for: CD4 count recovery and associated factors among individuals enrolled in the South African antiretroviral therapy programme: An analysis of national laboratory based data
Source: PLoS One. 2019 May 31;14(5):e0217742. doi: 10.1371/journal.pone.0217742 (PMC6544279; doi:10.1371/journal.pone.0217742)
Supplement: S2 Table — (DOCX) [file pone.0217742.s003.docx]

**S2 Table: Predicted CD4 counts (with confidence intervals) among those with or without viral suppression (N= 1 070 900)**

|  | **12 months** | **24 months** | **36 months** | **48 months** | **54 months** |
| --- | --- | --- | --- | --- | --- |
| <200_VLS | 267 (265- 268) | 327 (325- 329) | 359 (356- 362) | 371 (367- 376) | 376 (370- 382) |
| <200_NVLS | 201 (200- 203) | 248 (248- 246) | 290 (288- 293) | 291 (286- 296) | 318 (311- 324) |
| >=200_VLS | 481 (479- 483) | 514 (511- 517) | 544 (540- 548) | 576 (570- 581) | 585 (578- 591) |
| >=200_NVLS | 449 (447- 452) | 470 (467- 473) | 470 (466- 474) | 497 (491- 503) | 492 (486- 498) |

<200_VLS_p= mean predicted CD4 counts for individuals with baseline CD4 count < 200 cells/µl and had viral suppression during follow up; <200_NVLS_p=mean predicted CD4 counts for individuals with baseline CD4 count < 200 cells/µl and did not have viral suppression during follow up; >=200_VLS_p= mean predicted CD4 counts for individuals with baseline CD4 count >= 200 cells/µl and had viral suppression during follow up; >=200_NVLS_p = mean predicted CD4 counts for individuals with baseline CD4 count >=200 cells/µl and did not have viral suppression during follow up; lb= lower bound of the confidence interval; ub= upper bound of the confidence interval
