# Supplementary material for: CD4 count recovery and associated factors among individuals enrolled in the South African antiretroviral therapy programme: An analysis of national laboratory based data
Source: PLoS One. 2019 May 31;14(5):e0217742. doi: 10.1371/journal.pone.0217742 (PMC6544279; doi:10.1371/journal.pone.0217742)
Supplement: S3 Table — (DOCX) [file pone.0217742.s004.docx]

**S3 Table: Predicted CD4 count recovery among individuals aged 50 years or older at ART start (N= 324,677)**

| **CD4 count at the start of ART (cells/µl)** | **Duration on ART** | **Predicted CD4 count males**  **(cells/µl)** | **Predicted CD4 count females**  **(cells/µl)** |
| --- | --- | --- | --- |
| <50 | 12 months | 159 (158- 161) | 192 (190- 193) |
|  | 24 months | 213 (211- 215) | 262 (260- 264) |
|  | 36 months | 240 (238- 243) | 301 (298- 305) |
|  | 48 months | 254 (249- 258) | 323 (318- 328) |
|  | 54 months | 253 (245- 260) | 325 (318- 331) |
| 50- 199 | 12 months | 264 (263- 266) | 305 (303- 307) |
|  | 24 months | 305 (303- 307) | 364 (362- 366) |
|  | 36 months | 323 (321- 326) | 397 (394- 400) |
|  | 48 months | 334 (330- 338) | 418 (414- 422) |
|  | 54 months | 339 (334- 344) | 422 (417- 428) |
| 200- 349 | 12 months | 398 (396- 400) | 449 (447- 451) |
|  | 24 months | 426 (424- 429) | 495 (492- 497) |
|  | 36 months | 439 (435- 442) | 527 (524- 530) |
|  | 48 months | 466 (461- 471) | 553 (548- 558) |
|  | 54 months | 463 (457- 469) | 577 (572- 583) |
| 350- 499 | 12 months | 430 (428- 433) | 488 (485- 490) |
|  | 24 months | 455 (452- 458) | 527 (524- 530) |
|  | 36 months | 477 (472- 481) | 565 (561- 570) |
|  | 48 months | 495 (489- 501) | 593 (587- 599) |
|  | 54 months | 513 (506- 519) | 603 (597- 610) |
| >=500 | 12 months | 578 (575- 581) | 629 (626- 632) |
|  | 24 months | 582 (578- 586) | 662 (658- 665) |
|  | 36 months | 602 (597- 607) | 692 (687- 697) |
|  | 48 months | 573 (566- 580) | 699 (693- 706) |
|  | 54 months | 612 (606- 619) | 705 (697- 712) |
