# Supplementary material for: CD4 count recovery and associated factors among individuals enrolled in the South African antiretroviral therapy programme: An analysis of national laboratory based data
Source: PLoS One. 2019 May 31;14(5):e0217742. doi: 10.1371/journal.pone.0217742 (PMC6544279; doi:10.1371/journal.pone.0217742)
Supplement: S4 Table — (DOCX) [file pone.0217742.s005.docx]

**S4 Table: Predicted CD4 recovery at different durations on ART among males 15- 49 years by baseline CD4 count and province (N= 192 066)**

|  | **EC** | **FS** | **GP** | **KZN** | **LP** | **MP** | **NC** | **NW** | **WC** | **All** |
| --- | --- | --- | --- | --- | --- | --- | --- | --- | --- | --- |
| **50- 199 cells/µl** |  |  |  |  |  |  |  |  |  |  |
| 12 months | 257 (255- 258) | 272 (270- 274) | 258 (257- 260) | 260 (259- 261) | 274 (272- 276) | 257 (256- 259) | 266 (263- 267) | 265 (263- 267) | 268 (267- 270) | 262 (261- 264) |
| 36 months | 313 (310- 316) | 328 (324- 331) | 325 (322- 327) | 329 (327- 332) | 319 (316- 323) | 326 (323- 330) | 299 (294- 304) | 315 (311- 318) | 314 (311- 317) | 324 (322- 327) |
| 54 months | 314 (309- 319) | 344 (334- 354) | 351 (345- 356) | 351 (346- 356) | 315 (305- 324) | 321 (314- 330) | 274 (259- 289) | 320 (311- 328) | 321 (314- 328) | 337 (330- 344) |
| **≥200 cells/µl** |  |  |  |  |  |  |  |  |  |  |
| 12 months | 416 (414- 419) | 442 (439- 446) | 416 (414- 418) | 432 (430- 434) | 416 (413- 419) | 420 (418- 422) | 465 (461- 470) | 384 (381- 386) | 412 (410- 414) | 424 (422- 427) |
| 36 months | 418 (414- 422) | 434 (429- 439) | 460 (457- 464) | 486 (485- 489) | 436 (431- 440) | 448 (444- 453) | 437 (430- 443) | 477 (473- 482) | 444 (440- 447) | 470 (466- 473) |
| >=54 months | 454 (445- 464) | 486 (473- 499) | 504 (496- 512) | 516 (509- 524) | 475 (462- 488) | 462 (452- 472) | 394 (378- 410) | 460 (449- 471) | 457 (448- 466) | 497 (489- 506) |
