# Supplementary material for: CD4 count recovery and associated factors among individuals enrolled in the South African antiretroviral therapy programme: An analysis of national laboratory based data
Source: PLoS One. 2019 May 31;14(5):e0217742. doi: 10.1371/journal.pone.0217742 (PMC6544279; doi:10.1371/journal.pone.0217742)
Supplement: S5 Table — (DOCX) [file pone.0217742.s006.docx]

**S5 Table: Predicted CD4 recovery at different durations on ART among females 15- 49 years by baseline CD4 count and province (N=** **542 439)**

|  | **EC** | **FS** | **GP** | **KZN** | **LP** | **MP** | **NC** | **NW** | **WC** | **All** |
| --- | --- | --- | --- | --- | --- | --- | --- | --- | --- | --- |
| **50- 199 cells/µl** |  |  |  |  |  |  |  |  |  |  |
| 12 months | 309 (307- 311) | 323 (321- 325) | 304 (303- 305) | 305 (303- 306) | 303 (301- 305) | 307 (306- 309) | 298 (294- 301) | 302 (300- 304) | 318 (316- 320) | 308 (306- 310) |
| 36 months | 386 (383- 386) | 402 (398- 406) | 404 (402 - 407) | 405 (403- 407) | 396 (392- 400) | 392 (389- 395) | 379 (372- 385) | 391 (388- 395) | 398 (395- 403) | 400 (397- 403) |
| 54 months | 401 (393- 409) | 438 (427- 449) | 449 (443- 455) | 444 (440- 449) | 409 (399- 420) | 416 (408- 424) | 359 (342- 376) | 408 (399- 417) | 407 (403- 411) | 429 (422- 436) |
| **≥200 cells/µl** |  |  |  |  |  |  |  |  |  |  |
| 12 months | 484 (481- 486) | 520 (517- 523) | 483 (481- 485) | 494 (492- 496) | 495 (492- 497) | 487 (485- 490) | 506 (502- 511) | 507 (504- 510) | 486 (484- 489) | 495 (493- 497) |
| 36 months | 538 (534- 542) | 548 (543- 553) | 558 (554- 561) | 579 (575- 582) | 538 (533- 543) | 552 (548- 556) | 512 (504- 524) | 532 (527- 537) | 541 (538- 545) | 563 (559- 567) |
| 54 months | 559 (549- 570) | 596 (583- 609) | 614 (605- 622) | 628 (623– 634) | 567 (553- 580) | 579 (568- 590) | 502 (482- 522) | 565 (553- 577) | 561 (552- 571) | 609 (600- 617) |
