# Supplementary material for: CD4 count recovery and associated factors among individuals enrolled in the South African antiretroviral therapy programme: An analysis of national laboratory based data
Source: PLoS One. 2019 May 31;14(5):e0217742. doi: 10.1371/journal.pone.0217742 (PMC6544279; doi:10.1371/journal.pone.0217742)
Supplement: S6 Table — (DOCX) [file pone.0217742.s007.docx]

**S6 Table : Predicted CD4 counts at 12 months post ART initiation by baseline CD4 counts and calendar year of ART initiation (N=1 070 900)**

| **CD4 count at ART start** | **2010** | **2011** | **2012** | **2013** | **2014** |
| --- | --- | --- | --- | --- | --- |
| <50 cells/µl | 169 (168- 170) | 165 (164- 167) | 185 (184- 187) | 185 (184- 187) | 192 (190- 195) |
| 50-199 cells/µl | 279 (278- 281) | 274 (273- 276) | 300 (298- 301) | 305 (303- 306) | 299 (297- 302) |
| 200-349 cells/µl | 411 (409- 413) | 413 (412- 415) | 442 (440- 444) | 448 (446- 450) | 453 (450- 456) |
| 350-499 cells/µl | 454 (452- 456) | 456 (454- 458) | 485 (482- 487) | 498 (495- 500) | 501 (498- 505) |
| >500 cells/µl | 603 (600- 605) | 594 (591- 597) | 626 (623- 629) | 637 (634- 640) | 637 (633- 641) |
